# Supplementary material for: The discovery of novel noncoding RNAs in 50 bacterial genomes
Source: Nucleic Acids Res. 2024 Apr 22;52(9):5152–65. doi: 10.1093/nar/gkae248 (PMC11109978; doi:10.1093/nar/gkae248)
Supplement: gkae248_Supplemental_Files [file gkae248_supplemental_files.zip › Narunsky 50 Genomes RNA Motifs Files.zip!/Publication/45-O_oeni/Figures/LRC-45-2-NC_008528_1_IGR_8_802817_803120.sto.pdf]

LRC-45-2-NC_008528_1_IGR_8_802817_803120.sto.r2r

5′-○○YU●AUU●UCUCUUUUCUU●RRUUUUYRAAU●YRURAAUUAAU●UYUUACACAAYAAAGUAUG●UCUGAAARCAUAYYYYUGUCUAGCAACYAAR●CAUGAUAUYYCGCAYYAAUUAACCUAG●UUUGCGCAA●YUGYUUUU●CYUYGUU●●YCAAAA●●ACCG●U●YRAARG●YUUUAUCYGGUUGCGRRAGAURUU●RAUUYG●UGRU●GG●●●UUU●●A●A●UR●GYUUU●G●U●URA●UAU●●UUAA●CGAG●RA●U●●AUY●UA●U●GUCRYURA○○○○○○○○○○○○○○○○○○○○○○○
